# Realization of Hollow-Core Photonic-Crystal Fiber Optic Gyro Based on Low-Noise Multi-Frequency Lasers with Intermediate-Frequency Difference

**DOI:** 10.3390/s20102835

**Published:** 2020-05-16

**Authors:** Hongchen Jiao, Lishuang Feng, Qingjun Zhang, Jie Liu, Tao Wang, Ning Liu, Chunqi Zhang, Xindong Cui, Xiaoning Ji

**Affiliations:** 1 Beijing Institute of Spacecraft System Engineering, China Academy of Space Technology, Beijing 100094, China; ztzhangqj@163.com (Q.Z.); liujie5@spacechina.com (J.L.); 15210004024@163.com (T.W.); 2 Precision Opto-Mechatronics Technology, Key Laboratory of Education Ministry, Beihang University, Beijing 100191, China; fenglishuang@buaa.edu.cn (L.F.); ning.liu@buaa.edu.cn (N.L.); zhangchunqi@buaa.edu.cn (C.Z.); 3 Research Center of Satellite Technology, Harbin Institute of Technology, Harbin 150001, China; 4 Beijing Institute of Applied Meteorology, Beijing 100029, China; 17710428829@163.com; 5 Air Force Research Institute, Beijing 100000, China; 13220192564@139.com

**Keywords:** fiber optic sensor, resonator fiber optic gyro, fiber resonator, hollow-core photonic-crystal fiber, optical phase-locked loop

## Abstract

Mainly focusing on the demand for a novel resonator optic gyro based on a hollow-core photonic-crystal fiber (HC-RFOG), we achieve a multi-frequency lasers generation with low relative phase noise via an acousto-optic modulation of light from a single laser diode. We design a homologous heterodyne digital optical phase-locked loop (HHD-OPLL), based on which we realize the low-noise multi-frequency lasers (LNMFLs) with an intermediate frequency difference. The noise between the lasers with a 20 MHz difference is 0.036 Hz, within the bandwidth of 10 Hz, in a tuning range of 120 kHz, approximately 40 dB lower than that produced without the HHD-OPLL. Finally, based on the LNMFLs, an HC-RFOG is realized and a bias stability of 5.8 °/h is successfully demonstrated.

## 1. Introduction

Resonator fiber optic gyros (RFOGs) based on the hollow-core photonic-crystal fiber (HC-RFOGs), combining the miniaturization of RFOGs and the environmental stability of hollow-core photonic-crystal fibers (HCPCFs), are considered as potential alternates for navigation grade optic gyros [1,2,3]. As reported, the differential detection scheme is one of the most promising solutions for suppressing nonreciprocal noises in HC-RFOGs [4,5,6]. The generation of multiple-frequency lasers with low relative phase noise and radio wavelength frequency differences is the key requirement for the differential detection scheme [7,8]. For HC-RFOGs, the required frequency differences between the lasers are usually only in the order of tens of MHz [9,10,11].

The most widely used low-noise multi-frequency lasers generation method is the optical sideband modulation (OSBM) [12,13,14]. This method produces a series of optical sideband signals with a fixed frequency difference by an external modulation of the laser (amplitude modulation or phase modulation). These sideband signals have the same random phase. However, this method inevitably generates non-ideal side modes and a residual carrier signal, and there is a potential problem of intermodulation distortion. To obtain multi-frequency lasers with clean spectrums, researchers have proposed the optical injection locking (OIL) method [15], the optical phase-locked loop (OPLL) method [16], and the optical injection phase-locked loop (OIPLL) method [17]. However, the capacity for the suppression of low-frequency noise via the OIL method is seriously affected by the injection line and environmental factors. The OPLL method, using two independent lasers and a phase-locked loop to generate the beat signal, can further improve the low-frequency phase noise suppression capability. However, the OPLL method imposes stringent requirements on the loop delay and laser linewidth, and there are problems associated with the high-frequency oscillation and periodic loss of locking. The OIPLL method combines the advantages of both the OIL and OPLL methods, and relaxes the strict requirements of the system regarding the loop delay and laser linewidth. However, the structure needed to implement the OIPLL approach is more complicated and costlier.

In this paper, mainly focusing on the demand of the novel HC-RFOG, we suggest a method to generate low-noise multi-frequency lasers (LNMFL) with an intermediate frequency (IF) difference based on a single laser diode. In our method, a homologous heterodyne digital optical phase-locked loop (HHD-OPLL) replaces the slave laser and the injection locking module, respectively, in a conventional OIPLL system. While inheriting the advantages of the OIPLL technique, as shown in Table 1, our approach simplifies the structure of the system.

Hence, the technique we present should have significance for applications in the field of HC-RFOGs. Based on the above-described development, we obtained a 0.036 Hz beat signal noise, with an integration bandwidth of 10 Hz, for multi-frequency lasers with a 20 MHz difference and a tuning range of about 120 kHz. As calculated, with this approach, the noise of the HC-RFOG introduced by the lasers could be reduced to lower than 0.05 °/h with an integration time 10 s, and a measurement range of about 100 °/s should be realized. Finally, we realized an HC-RFOG based on the LNMFLs, and a bias stability of 5.8 °/h was successfully demonstrated.

## 2. Methods

### 2.1. Overall Structure

**Figure 1:**
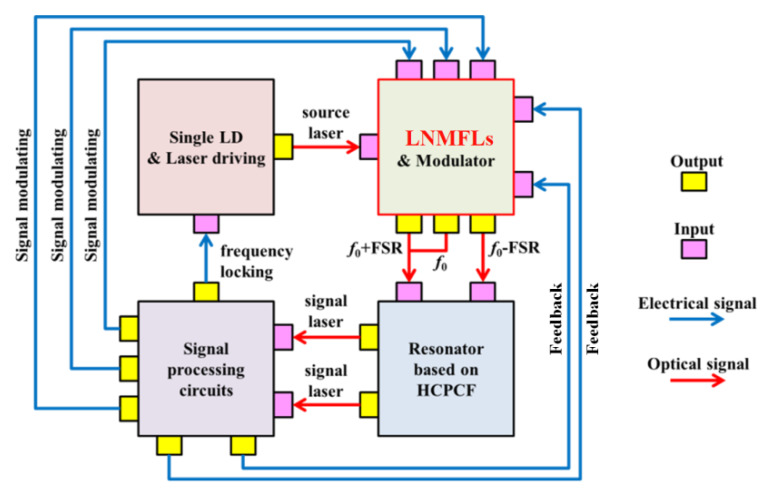
Topological structure of the HC-RFOG.

The LNMFL generation method in this paper mainly focuses on the demand of the HC-RFOG. We designed an HC-RFOG [5], and we adopted multi-frequency lasers to suppress the laser frequency noises of the gyro in this paper. The frequency difference between the lasers is an integral multiple of the free spectrum range (FSR) of the fiber resonator. In our design, the FSR is about 20 MHz. With referencing to the FSR of the fiber resonator in our design, all the frequency differences between the multi-frequency lasers in the following experiments were set to 20 MHz. The topological structure of the HC-RFOG is shown in Figure 1.

The HC-RFOG consists of a single laser diode (LD) and its driving, a group of LNMFL modules, some modulators, a resonator based on a hollow-core photonic-crystal fiber (HCPCF), and a set of signal processing circuits. The signal transmission process in the HC-RFOG is shown in Figure 1. For the HC-RFOG, the sensitivity is proportional to the noise between the lasers.

**Figure 2:**
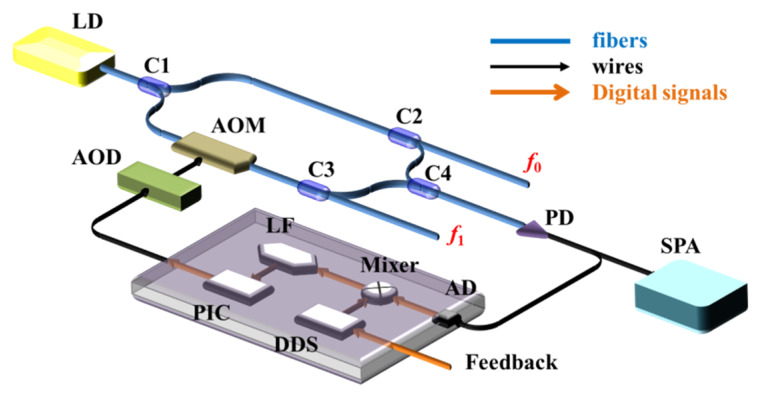
Low-noise multi-frequency lasers generation system.

We designed and implemented the LNMFL generation system as shown in Figure 2. This system includes a laser diode (LD), four splitters (C1, C2, C3, C4), an acousto-optic modulator (AOM) with an acousto-optic drive (AOD), a photodetector (PD), a spectrum analyzer (SPA), and a digital processing module. The digital processing module includes an analog–digital converter (AD), mixer, direct digital synthesizer (DDS), loop filter (LF), and PI controller (PIC).

The laser from the LD is divided into two parts after C1: the eigenfrequency laser (EFL) and the up-converted laser (UCL). The EFL, which is unmodulated, forms the signal light component of the frequency (*f*_0_). The UCL, modulated by the AOM, forms the signal light component of the frequency (*f*_1_). The frequency difference Δ*f* = *f*_0_ − *f*_1_ between the EFL and UCL is determined by the frequency of the driving signal applied to the AOM by the AOD. Parts of the EFL and UCL are extracted through C2 and C3 and mixed at C4 to form an optical beat signal. This optical beat signal is converted into an electrical oscillation after being detected by a photodiode (PD), and a component of this is introduced into the SPA to observe its spectrum. The remainder of the analog signal from the PD is converted into a digital signal by the AD and introduced to a field-programmable gate array (FPGA). This digital signal is mixed with the local oscillator signal (*f_r_*) (reference signal, generated by the DDS in the FPGA, and could be modulated by the feedback signal shown in Figure 1) to derive two signals with frequencies of Δ*f* − *f_r_* and Δ*f* + *f_r_*. The component with the frequency of Δ*f* − *f_r_* represents the phase difference between the optical beat signal and the reference signal. By using the LF to filter out the signal with the frequency of Δ*f* + *f_r_*, the phase difference is obtained, and this signal is sent to the PIC to control the UCL. Finally, the frequency difference between *f*_0_ and *f*_1_ is locked to the frequency of the reference signal. In this arrangement, the phase noise between the EFL and the UCL is also locked.

**Figure 3:**
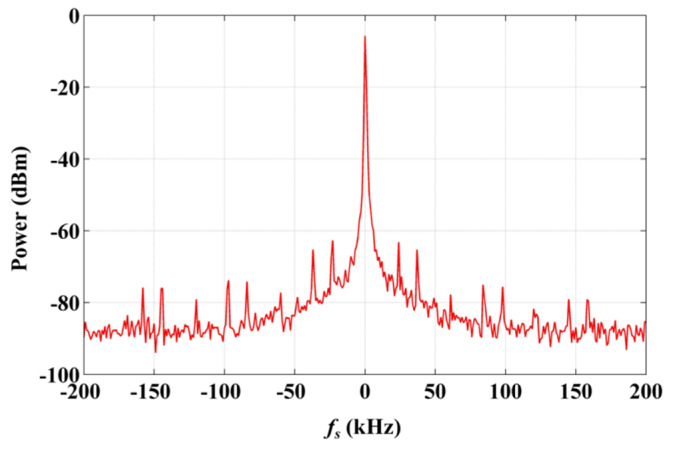
Spectrum of the signal of beat between the eigenfrequency laser (EFL) and the up-converted laser (UCL), acquired without using the optical phase-locked loop (OPLL).

It is an available and convenient method to realize multi-frequency lasers based on AOMs. The common mode noise between these lasers can be cancelled out, as the phases of them are consistent. We recorded the signal of the beat between these homologous lasers with an IF difference, without using the OPLL, and the result is shown in Figure 3 (100 Hz video bandwidth, 1 Hz resolution bandwidth), in which the *f*_s_ is the frequency offset with respect to 20 MHz. The linewidth of the beat signal is 500 Hz, which is much smaller than that of the system based on lasers from different LDs (about 10 kHz).

Although the use of the AOM results in the linewidth of the beat signal being improved by two orders of magnitude, this method alone cannot meet the needs of high-resolution systems. For example, multi-laser beat linewidths typically need to be reduced to the Hz or even mHz level to meet the accuracy requirements for advanced HC-RFOGs. The main factor determining the beat linewidth is the phase noise introduced by the AOM and AOD. Therefore, an optimized OPLL is needed to further suppress the relative phase noise from the multi-frequency lasers.

### 2.2. Homologous Heterodyne Digital Optical Phase-Locked Loop

**Figure 4:**
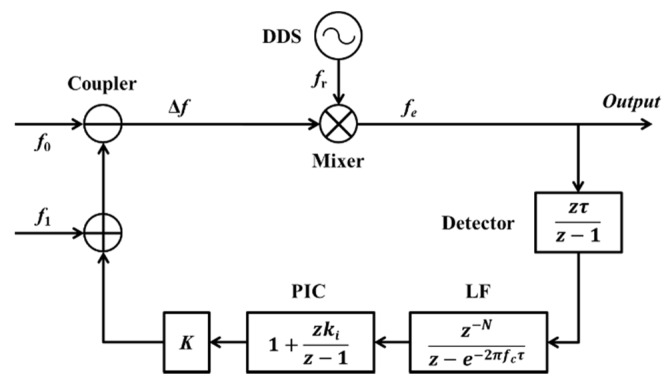
Small-signal model of the HHD-OPLL.

The signal flow of the small-signal model of the homologous heterodyne digital optical phase-locked loop (HHD-OPLL) in the discrete domain is shown in Figure 4. *f_e_* is the error between the Δ*f* and *f_r_*, *τ* is the clock of the digital system, and *f*_c_ is the bandwidth of the LF. *N* is the number of registers in the loop (corresponding to a loop delay (*τ_d_*)). The gain of the integral link relative to that of the proportional link is *k_i_*, which is usually much smaller than one. The total loop gain is *K*, which can be derived from the loop stability condition.

The transfer function from the *f_e_* to the output of the HHD-OPLL is

(1) $$H_{n}=\frac{1}{1+\frac{z\tau }{z-1}\frac{z^{-N}}{z-e^{-2\pi f_{c}\tau }}\left(1+k_{i}\frac{z\tau }{z-1}\right)K}$$

As the relative frequency noise of the EFL and UCL is much bigger than the noise of the DDS in practice, the *f_e_* has a power spectral density (PSD) (*S_fe_*(*f*)) equivalent to that of white noise, and the amplitude of the PSD is equal to the initial beat linewidth (δ*f*). Thus, corresponding to this frequency noise, the phase fluctuation variance (*σ*^2^) of the beat signal after locking can be expressed as

(2) $$\begin{array}{c} \sigma^{2}=\int_{0}^{bandwidth}S_{fe}\left(f\right){\left|H_{n}/f\right|}^{2}df\\ S_{fe}\left(f\right)=\delta f \end{array}$$

The mean time between cycle slips (*T_ll_*) can be expressed as [19,20]

(3) $$\begin{array}{c} T_{ll}=\pi \frac{e^{\left(\frac{2}{\sigma^{2}}\right)}}{4B_{n}}\\ B_{n}=\int_{0}^{f_{c}}{\left|1-H_{n}\right|}^{2}df \end{array}$$

As the *T_ll_* is set to 24 h, the relationship between the loop delay (*τ_d_*), the loop bandwidth (*f_c_*), and the initial beat linewidth (δ*f*) can be expressed as

(4) $$\begin{array}{c} \delta f\leq \frac{\pi }{I_{p}}\frac{2}{\ln\left(T_{ll}4B_{n}/\pi \right)}\\ I_{p}=\int_{0}^{f_{c}}{\left|H_{n}/f\right|}^{2}df \end{array}$$

Based on the actual system, the *τ* is set to 1 × 10^−8^, the *N* is set to 10 (corresponding to a *τ_d_* of 100 ns), and the *k_i_* is set to 0.001. In addition, to achieve an optimal filtering performance while taking advantage of the bandwidth of the AOD, the *f_c_* is set to 100 kHz.

According to the analysis above, as δ*f* for the homologous lasers based on the AOM approach is 500 Hz and *f_c_* is 100 kHz, *τ_d_* for the HHD-OPLL should be less than 7 μs (the N should be less than 700). In contrast, as the value of δ*f* for two LDs is about 10 kHz and *f_c_* in the same case is about 3 MHz, the corresponding value of *τ_d_* should be less than 300 ns (the N should be less than 30). Hence, it is apparent that by using the AOM-based HHD-OPLL method, the stringent requirements of phase-locked loop systems for bandwidth and delay are avoided. This result theoretically verifies the advantages of this LNMFL generation scheme based on an HHD-OPLL as detailed in this paper.

**Figure 5:**
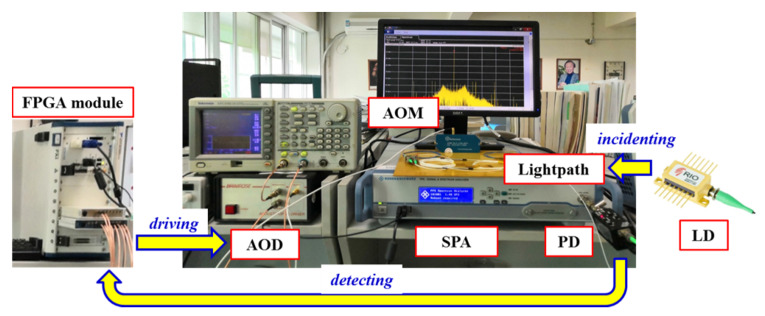
Structure of the test system for the low-noise multi-frequency laser (LNMFL) based on the HHD-OPLL.

The test system for the LNMFL based on the HHD-OPLL is realized as shown in Figure 5, and the mature instruments are adopted as a prototype. This system consists of an AOM and its AOD (Brimrose, AMF-20-2-1550, with a tuning bandwidth ≥ 100 kHz and a tuning range ≥ 2MHz), an SPA (ROHDE & SCHWARZ, FPS), an LD (RIO, PLANEX, with a maximum output power of 10 mW), a PD (Terahertz Technologies Inc., Shenzhen, China, with a maximum detection light power ≥ 10mW and a detection bandwidth ≥ 100MHz (five times the beat signal frequency)), an FPGA module (NI, PXIe-1082), and a set of lightpaths including three normal fiber couplers (corresponding to an insertion loss ≥ 9dB).

The polarization axis of the LD output fiber and the polarization axis of the AOM input fiber are accurately aligned with a polarization-maintaining fusion splicer, and the polarization crosstalk is less than −40dB. At the same time, the initial polarization extinction ratio of the output light of the selected LD is better than 20 dB, which can well meet the requirements of the AOM.

In our further research, to realize a modularized and miniaturized LNMFL system, the signal processing circuits would be designed as exclusive and integrated, and the lightpath would be realized by optical microstructures and integrated optical waveguides.

**Figure 6:**
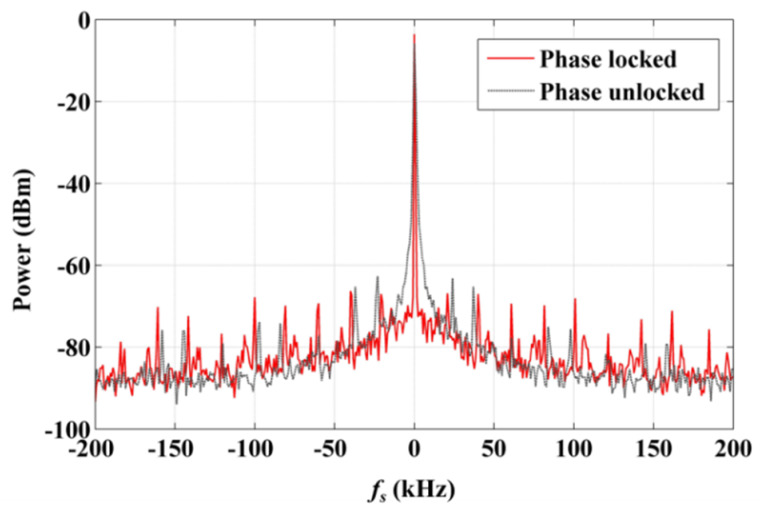
Spectra of EFL and UCL beat signal obtained using OPLL, with phase locking and without phase locking.

The test results of the beat signals, with this HHD-OPLL, are shown in Figure 6 (100 Hz video bandwidth, 1 Hz resolution bandwidth). The gray line indicates the spectral distribution of the beat signal with the phase unlocked, and the red line marks the spectral distribution of the beat signal with the phase locked. The linewidth of the phase-locked beat signal is less than the resolution bandwidth limit of the SPA (<1 Hz). It can be seen that the beat linewidth is dramatically narrowed upon switching on the phase locking, which also means that the relative phase between the EFL and UCL is locked and the relative phase noise is suppressed in this case. At the same time, the beat signal energy is increased, and this change is attributed to the energy of the noise within the lock-in bandwidth being partially transferred to the beat signal. The side peaks in Figure 6 were introduced because of the inevitable weak impedance mismatch at the circuit connection points where the undesired harmonic signals are generated, modulating the light through the AOM. In this system, the side mode suppression amount is greater than 60 dB.

## 3. Results and Discussion

**Figure 7:**
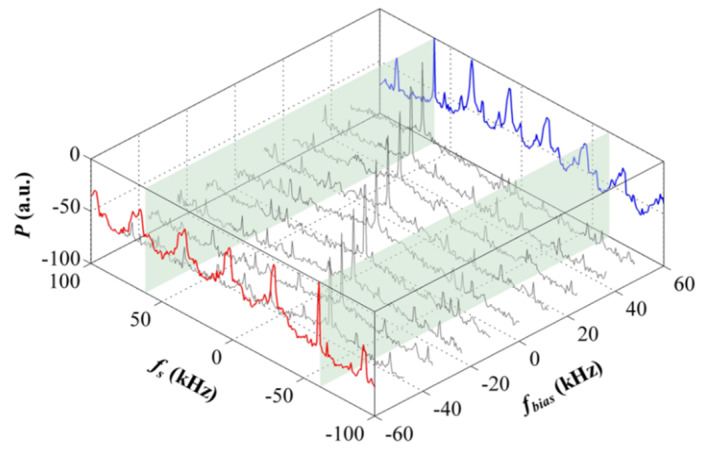
Spectra of beat signals locked with different frequency bias values.

A series of experiments to verify the LNMFL generation scheme were completed. In order to verify the tunability and tuning range of the system, we changed the DDS frequency via the digital interface to achieve continuous variation of the beat signal within a certain range. The result is shown in Figure 7 (100 Hz video bandwidth, 1 Hz resolution bandwidth). When the frequency bias (*f*_bias_) (the frequency offset with respect to 20 MHz) goes beyond the pull-in range, the HHD-OPLL ceases to be locked, as shown by the −60 kHz (blue) and +60 kHz (red) lines in Figure 7. Hence, it can be seen that the pull-in range of the HHD-OPLL (that is the tuning range of the system) is 120 kHz, which is 0.6% of the carrier frequency. This tuning range corresponds to a measurement range of about 100 °/s, which can satisfy the requirement of most of the RFOGs in their application.

**Figure 8:**
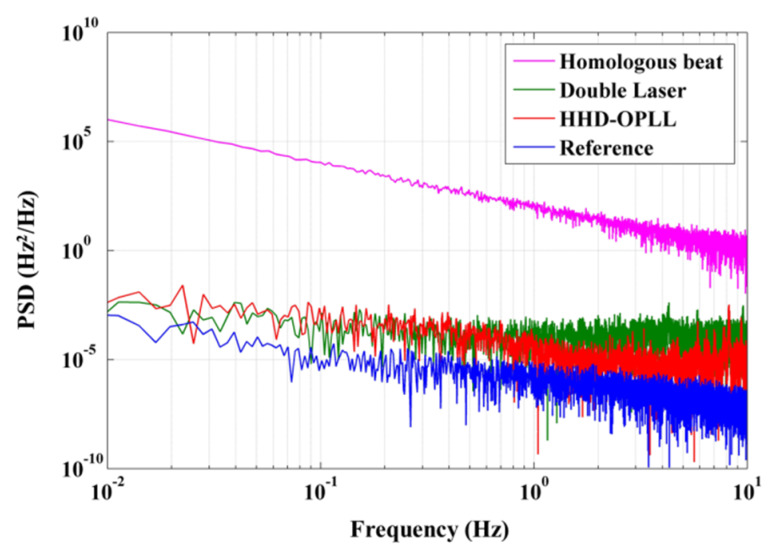
Power spectral densities (PSDs) of beat frequency noises under various conditions.

In order to verify the advantages of the LNMFL generation system based on the HHD-OPLL, we performed beat tests using various methods (with homologous multi-frequency lasers generated by AOMs, double lasers, multi-frequency lasers based on the HHD-OPLL, and the reference signal generated by the DDS in the FPGA, respectively) and calculated the PSDs of the beat frequency noises in the bandwidth of 10 Hz (corresponding to the sampling bandwidth of RFOGs). The results are shown in Figure 8.

The magenta line is the frequency noise of the beat signal of the homologous lasers without the OPLL, and the standard deviation of the frequency noise of this beat signal is 189 Hz, with an integration bandwidth of 10 Hz. In contrast, the red line is the frequency noise of the beat signal of the homologous lasers with the OPLL, and the standard deviation of the frequency noise of this beat signal is 0.036 Hz, with the same integration bandwidth. Hence, by locking the relative phase noise between the EFL and UCL, the frequency noise of the beat signal was reduced by nearly four orders of magnitude. Compared with the method based on two independent lasers and OPLLs (green line, Figure 8), the HHD-OPLL method presented in this paper has the same low-frequency noise characteristics and lower noise in the higher frequency band. This indicates that the influence of low-frequency noise introduced by environmental factors is the same for the different systems, but the high-frequency noise, principally random phase noise, is mechanically suppressed thanks to the use of the heterodyne digital optical phase-locked loop. In addition, we tested the frequency noise of the DDS as the blue line in Figure 8, and the standard deviation of the frequency noise of the DDS is 0.006 Hz with an integration bandwidth of 10 Hz: this can be treated as the noise limit of the HHD-OPLL.

**Figure 9:**
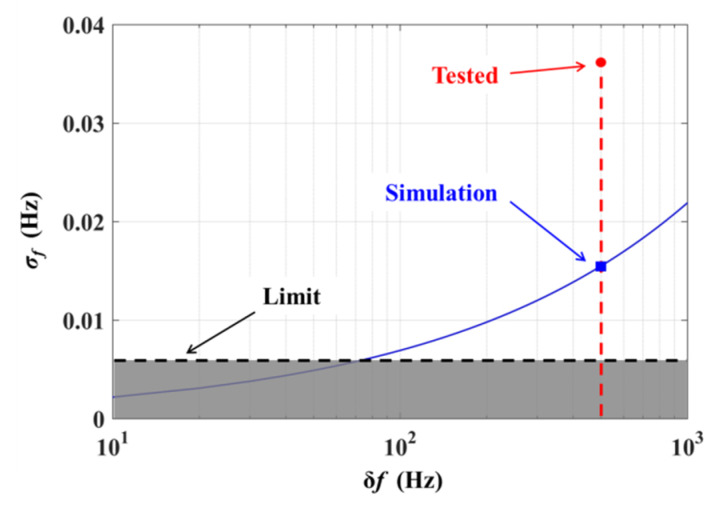
Frequency noise of beat signals as a function of the initial beat linewidth.

We estimated the frequency noise of the beat signal introduced by the different initial beat linewidths, and the result is shown in Figure 9 as the blue line. It can be seen that a larger value of δ*f* results in a deterioration of the frequency noise. As the experimental-measured δ*f* is 500 Hz, corresponding to the red dotted line in Figure 9, the theoretical simulation value of the beat frequency noise is 0.016 Hz (blue rectangle, Figure 9). However, the experimental beat frequency noise is 0.036 Hz (red dot, Figure 9), a little greater than the value obtained by the simulation, owing to the fluctuations in the environmental parameters (temperature, stress, etc.) related to the optical path.

As calculated, the relative frequency noise is a factor of only approximately 2 × 10^−9^ of the IF carrier and 2 × 10^−16^ of the optical carrier. It can be seen that there is still much room for improvement in the homologous laser beat linewidth and the environmental stability of the optical path. In addition, when the beat linewidth can be further reduced (less than 70 Hz), the frequency noise will reach the limit of 0.006 Hz, as shown by the black dashed line in Figure 9. In this case, the sensitivity of the HC-RFOG can reach 0.01 °/h and even better, which indicates a remarkable prospect for the HC-RFOG based on the LNMFL system.

**Figure 10:**
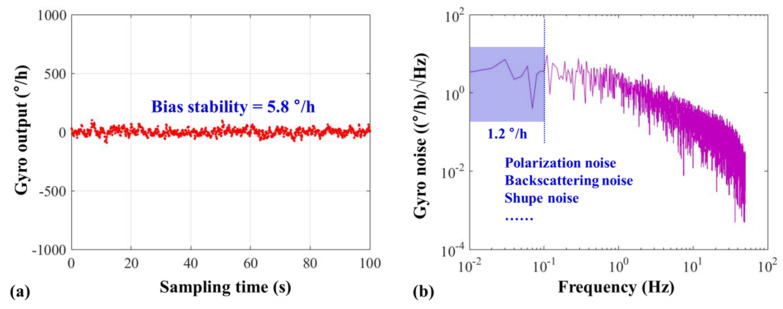
Test result of the HC-RFOG based on LNMFLs. (**a**) Data of the HC-RFOG with a sampling bandwidth of 10 Hz. (**b**) Spectral distribution of the noise of the HC-RFOG.

Based on the LNMFLs designed in this paper, we realized an HC-RFOG with multi-frequency differential detection, and the test result is shown as Figure 10a. The bias stability of the HC-RFOG is 5.8 °/h with an integration time of 10 s (corresponding to the normal output bandwidth of 0.1 Hz of RFOGs [21]). As shown in Figure 10b, the spectrum analysis of the gyro output shows that the gyro noise in the 0.1 Hz bandwidth is 1.2 °/h. However, calculated with Figure 8, the noise introduced by the LNMFL is only about 0.017Hz, with an integration bandwidth of 0.1 Hz. This is equivalent to a gyro noise of 0.05 °/h, about two orders of magnitude lower than the test result. This is because in addition to the laser frequency noise in the HC-RFOG, there are also polarization noise, backscatter noise, and shupe noise, and their effects are highlighted as the laser frequency noise is suppressed by the LNMFLs.

## 4. Conclusions

In order to reduce the laser frequency noise in the HC-RFOG, we presented a method to generate the LNMFL based on the AOM and HHD-OPLL. The homologous laser is generated by an acousto-optic frequency shifting, and the initial beat linewidth is only 500 Hz, which is nearly two orders of magnitude lower than the beat linewidth produced by the method employing independent LDs. On this basis, we reduced the frequency noise between the homologous lasers by four orders of magnitude through the use of an HHD-OPLL. The resultant noise was shown to be 0.036 Hz, under the 10 Hz bandwidth operating condition and the 20 MHz difference between the multi-frequency lasers, in a tuning range of 120 kHz. As the integration time is set to 10 s, this noise will only introduce a measurement noise of about 0.05 °/h in the HC-RFOG, indicating a remarkable prospect for novel RFOGs using the LNMFL system. Finally, based on the LNMFL designed in this paper, we realized an HC-RFOG with multi-frequency differential detection, and a bias stability of 5.8 °/h was successfully demonstrated with an integration time of 10 s.

Considering the design in this study, upon further optimizing the impedance matching of the loop and diminishing its environmental sensitivity, if the initial beat linewidth could be less than 70 Hz and the influence of environmental factors could be effectively reduced, a frequency noise between the multi-frequency lasers close to 0.006 Hz is expected to be realized. Combining the LNMFL with our research on polarization noise, backscattering noise, and shupe noise, an HC-RFOG system with a navigation level accuracy is expected to be implemented. These aspects are currently under investigation, and the relevant results will be published in subsequent articles.

## Figures and Tables

**Table 1:** Comparison of the optical injection phase-locked loop (OIPLL) and the homologous heterodyne digital optical phase-locked loop (HHD-OPLL).

Method	Number of LD	Noise Level	Cost (*A*)
OIPLL	Determined by the number of lasers *N* ≥ 2	0.032 Hz [18]	*N* × *A*
HHD-OPLL	1	0.036 Hz	*A*
